# Immunoinformatics‐Based Multi‐Epitope Vaccine Targeting *Helicobacter Pylori*

**DOI:** 10.1002/cnr2.70441

**Published:** 2025-12-28

**Authors:** Aytak Vahdat Khajeh Pasha, Mohammad Esfandiyari, Alireza Parnian, Mohammad Mahboubi‐Rabbani, Maryam Bayanati

**Affiliations:** ^1^ Department of Medicinal Chemistry, TeMS.C. Islamic Azad University Tehran Iran; ^2^ Student Research Committee Shiraz University of Medical Sciences Shiraz Iran; ^3^ Phytochemistry Research Center Shahid Beheshti University of Medical Sciences Tehran Iran

**Keywords:** gastric cancer, gene sequences, *Helicobacter Pylori*, immunodominant epitopes, immunogenicity, immunoinformatics, molecular docking, vaccine

## Abstract

**Background:**

The rising global incidence of *Helicobacter pylori* ‐related diseases, particularly gastric cancer, underscores the urgent need for effective preventive vaccines, motivating the exploration of innovative immunoinformatic strategies to address this public health challenge.

**Aims:**

The aim of this study was to design a multi‐epitope subunit vaccine for Helicobacter pylori using an immunoinformatics approach. Specifically, the objectives were to predict potential epitopes from the flagellin B and urease B proteins, integrate the cholera toxin B subunit (CTB) as a mucosal adjuvant, and perform computational validation of the vaccine construct for antigenicity, stability, and interaction with immune receptors.

**Methods:**

This study utilized an immunoinformatics approach to design a multi‐epitope subunit vaccine, involving epitope prediction from flagellin B and urease B, integration of the cholera toxin B subunit (CTB) as a mucosal adjuvant, and computational validation through tools like VaxiJen, Phyre2, MolProbity, and HDOCK for antigenicity, structure, and docking analysis.

**Results:**

The resulting vaccine construct comprises 406 amino acids with a molecular weight of 43 424.77 Da, exhibiting a predicted antigenic score of 1.0084, non‐allergenic and non‐toxic properties, and a stable physiochemical profile (instability index 23.51, GRAVY −0.425). Structural analysis suggested 99.1% (525/530) of residues in favored Ramachandran regions and 100.0% in allowed regions. Molecular docking with Toll‐like receptor 5 (TLR5) indicated a superior docking score of −309.05 and a confidence score of 0.9601, outperforming TLR2 (−250.74), with 10 CTL epitopes (6 from flagellin B, 4 from urease B), 6 HTL epitopes, and 2 LBL epitopes linked by AAY, GPGPG, and KK linkers, respectively.

**Conclusion:**

This research provides a computationally optimized vaccine design that shows potential for eliciting immune responses against *H. pylori* . Importantly, the findings remain entirely theoretical and require rigorous experimental validation in vitro and in vivo to assess their immunological relevance, safety, and efficacy before any translational or clinical application can be considered.

## 1 Introduction

Gastric cancer (GC) continues to be a public health challenge worldwide as the fifth most prevalent cancer and fourth most significant cause of oncological mortality [1]. GC accounts for approximately 7% of all cancers, resulting in over 800 000 deaths annually [2]. The worldwide occurrence has risen from 67 to 206 cases per 100 000 person‐years from 2005 to 2022 [3]. It occurs much more frequently in males, with an estimated 2:1 ratio compared to females [3]. *H. pylori* affects about 50% of the world's population and is a significant contributor to the development of gastric cancer, which is the second most common cause of cancer death globally [4]. *H. pylori* evokes chronic inflammation, which may progress to atrophic gastritis, intestinal metaplasia, dysplasia, and ultimately, gastric cancer [5].

Elimination of *H. pylori* has proven to reduce gastric cancer incidence, especially in those with precancerous lesions [6]. The escalation of antibiotic resistance, clarithromycin, and especially levofloxacin negatively impacts the effectiveness of treatments [7]. Standard triple therapy often does not yield satisfactory results, with 46% of cases demonstrating suboptimal outcomes [8]. Vaccine development for *H. pylori* has faced significant challenges. Poor biological understanding of *H. pylori* and the presence of antibiotic resistance have hindered efforts to develop an effective vaccine.

*H. pylori* possess varying virulence factors, such as urease, flagella, and outer membrane proteins, and it is difficult to determine which antigens would be effective for vaccination [9]. Flagella are important for adherence to gastrointestinal mucosa and result from dimerization, where flagellin B (FlaB) dimerizes with flagellin A (FlaA) [10]. The FlaA is considered the principal flagellin, with an extremely conserved nucleotide sequence that is highly immunogenic [10]. It has been shown that TLR5 recognizes flagellin from *H. pylori* [11]. Additionally, urease is a major colonization and virulence factor of *H. pylori* on the bacterial surface. Its virulent role is associated with hydrolysis of urea, producing carbon dioxide and ammonia, thus leading to changes in the surrounding pH [12]. Urease consists of two subunits, UreA and UreB, in the form of a hexamer. UreB enhances CD4+ and CD8+ T cell responses and offers protection against *H. pylori* [13].

Although research utilizing animal models has shown promise, no *H. pylori* vaccination has ever advanced to clinical testing in humans or been commercially available [14]. Factors such as the necessity for efficient adjuvants and delivery mechanisms have made the development process more complex [14].

Immunoinformatics presents a promising approach for designing subunit vaccines by employing computational tools to predict and analyze epitopes. In this study, we applied an immunoinformatics strategy to theoretically design a multi‐epitope subunit vaccine candidate for *H. pylori* , focusing on the particular importance of the microorganism in developing gastric cancer.

## 2 Materials and Methods

Figure S1 presents a concise schematic overview of the methodological framework employed in this study, detailing the in silico design of a multi‐epitope vaccine targeting *H. pylori* . This visual representation illustrates the sequential computational steps and tools utilized, including antigen selection, integration of the cholera toxin B subunit (CTB) as an adjuvant, and evaluation of open reading frame (ORF) sequences. The diagram serves as a comprehensive guide to the experimental procedures, ensuring a systematic approach to vaccine construct optimization and validation.

### 2.1 Genome Sequence Retrieval and Open Reading Frame (ORF) Identification

The genome of *Helicobacter pylori* strain CHC155 (NCBI Reference Sequence: NZ_AP026446.1) was retrieved from the NCBI genome database (https://www.ncbi.nlm.nih.gov/). The genome was downloaded in FASTA format to support subsequent analyses [15].

ORFs were identified using the ORF Finder tool (https://www.ncbi.nlm.nih.gov/orffinder/). The analysis employed standard parameters, and the output provided a compilation of possible protein‐coding areas for further examination [15].

### 2.2 Subcellular Localization Prediction

The PSORTb v3.0 tool, accessible at https://www.psort.org/psortb/, was used to predict the subcellular localization of the proteins encoded by the identified ORFs [16].

### 2.3 Signal Peptide Analysis

Signal peptides were identified using the SignalP v5.0 server, accessible at https://services.healthtech.dtu.dk/services/SignalP‐5.0/. Proteins with signal peptides were processed to remove these regions [17].

### 2.4 Transmembrane Helix Filtering

Proteins containing transmembrane helices were identified using the TMHMM v2.0 server, accessible at https://services.healthtech.dtu.dk/services/TMHMM‐2.0/ [18].

### 2.5 ORF Similarity Search With Antigens Using BLAST

The homology of ORF with known *H. pylori* antigens was investigated using the BLAST tool from the NCBI server [19], accessible at https://blast.ncbi.nlm.nih.gov/Blast.cgi?PROGRAM=blastp&PAGE_TYPE=BlastSearch&LINK_LOC=blasthome. The analysis employed the blast (Protein BLAST) algorithm with the nr (non‐redundant protein sequences) database [20]. The search was tailored to *H. pylori* as the target organism, using the E‐value as a metric for sequence alignment significance. This approach aimed to assess the antigenic potential of selected ORF for multi‐epitope vaccine design [21].

### 2.6 Epitope Prediction

Linear B lymphocyte (LBL) epitopes were determined using the tools available at the Immune Epitope Database (IEDB) [22], located at http://tools.iedb.org/bcell/.

Cytotoxic T lymphocyte (CTL) epitopes were recognized through the MHC‐I prediction tool provided by the Immune Epitope Database (IEDB) [23], accessible at http://tools.iedb.org/mhci/.

Helper T lymphocyte (HTL) epitopes were identified using the MHC class II binding allele prediction tool from the Immune Epitope Database (IEDB) [24], available at http://tools.iedb.org/mhcii/.

All predictions were performed using the default parameters of the servers. For each category, toxicity, antigenicity, and allergenicity were evaluated using the VaxiJen [25], AllerTOP [26], and ToxinPred [27] servers.

### 2.7 Multi‐Epitope Subunit Vaccine Construction

Selected epitopes were linked using appropriate linkers: AAY for cytotoxic T lymphocyte (CTL) epitopes, GPGPG for helper T lymphocyte (HTL) epitopes, and KK for linear B lymphocyte (LBL) epitopes. The entire cholera toxin B subunit (CTB) protein was incorporated as an adjuvant, connected using an EAAAK linker to enhance the immune response [28].

### 2.8 Antigenicity, Allergenicity and Toxicity Analysis

The antigenic potential of the proteins was evaluated through the VaxiJen v3.0 server (https://www.ddg‐pharmfac.net/vaxijen/VaxiJen/VaxiJen.html) [25], using a threshold value set at 0.4. In addition, the proteins were examined for allergenic properties using AllerTop v2.1 (https://www.ddg‐pharmfac.net/AllerTOP/) [26] and for toxicity via ToxinPred (http://crdd.osdd.net/raghava/toxinpred/) [27]. Only proteins that were non‐toxic, non‐allergenic, and possessed high antigenicity were selected for epitope prediction.

### 2.9 BLAST Homology Analysis

The final vaccine sequence was subjected to a BLAST search at https://blast.ncbi.nlm.nih.gov/Blast.cgi to ensure no significant homology with human or animal proteins. This step minimized the risk of autoimmunity or cross‐reactivity in the host [19].

### 2.10 Physiochemical Characterization

ProtParam, available at https://web.expasy.org/protparam/, was used to analyze the molecular weight, isoelectric point, stability index, and hydrophobicity of the vaccine construct [29].

### 2.11 Two‐Dimensional (2D) Structure Determination of Vaccine Construct

The two‐dimensional (2D) structure properties, including α‐helix, β‐strand, and random coils, of the designed protein were predicted using the Self‐Optimized Prediction Method with Alignment (SOPMA) server available at https://npsa‐pbil.ibcp.fr/cgi‐bin/npsa_automat.pl?page=/NPSA/npsa_mlrc.html under default parameters [30]. The MLRC analysis was performed using GOR4, SIMPA96, and SOPMA methods.

### 2.12 Three‐Dimensional (3D) Structure Determination of Vaccine Construct

The three‐dimensional structure of the protein was modeled using the Phyre2.2 server located at https://www.sbg.bio.ic.ac.uk/phyre2/html/page.cgi?id=index [31]. The predicted structure demonstrated similarity to the 7NST protein structure from the Protein Data Bank (PDB) (https://www.rcsb.org/) [32]. Structural validation and refinement were conducted using the MolProbity server to assess geometry, rotamer quality, and other structural parameters (http://molprobity.biochem.duke.edu/) [33]. The MolProbity analysis included metrics such as Ramachandran plot statistics, bond angles, and rotamer quality to ensure the model's reliability for downstream analyses.

### 2.13 Molecular Docking Exploration of Constructed Vaccine and Toll‐Like Receptor 5 (TLR5)

Molecular docking analyses were conducted using the HDOCK server (http://hdock.phys.hust.edu.cn), where the refined vaccine was utilized as the ligand and the Toll‐like receptor 5 (TLR5) protein served as the receptor [34, 35]. The TLR5 receptor plays a crucial role in innate immune responses and cytokine release [36]. The crystal structure of the TLR5 receptor (PDB ID: 3J0A) was obtained from the Protein Data Bank.

## 3 Result

### 3.1 Genome and ORFs Identification

The complete chromosomal genome of *H. pylori* strain CHC155 was retrieved from the National Center for Biotechnology Information (NCBI) GenBank database (Accession number: NZ_AP026446.1). The sequence, containing 1 720 840 base pairs, was downloaded in FASTA format and used for further bioinformatic analysis. To identify potential open reading frames (ORFs), the ORF Finder tool available on the NCBI website was employed. ORF Finder is a computational tool that identifies all possible ORFs in a given nucleotide sequence by scanning for start and stop codons. Using this tool, a total of 8254 ORFs were identified in the *H. pylori* genome.

### 3.2 Subcellular Localization

To predict the subcellular localization of the multi‐epitope subunit vaccine candidate, we utilized PSORTb, a reliable tool designed for the prediction of bacterial protein localization. Only proteins localized to the outer membrane and extracellular regions were selected, resulting in 28 extracellular and 37 outer membrane proteins for further study.

### 3.3 Signal Peptide

To refine the list of open reading frames (ORFs) for subsequent protein structure predictions, the presence of signal peptides was assessed. Signal peptides are short sequences that direct the nascent protein to specific cellular locations but are typically cleaved off during post‐translational modifications (PTMs). Therefore, it is crucial to exclude these sequences from structural predictions to ensure accuracy. Using the SignalP tool, all identified ORFs were analyzed to detect signal peptides. Among the extracellular proteins, 28 ORFs were identified, out of which 13 contained signal peptides, and 15 did not. Similarly, for the outer membrane proteins, 37 ORFs were analyzed, and 29 were found to contain signal peptides, while 8 did not. To proceed, the signal peptide sequences were removed from the corresponding ORFs. This ensures that only the mature protein sequences are carried forward for structural predictions and subsequent steps in the vaccine design process. By focusing on these refined sequences, we aim to enhance the reliability of downstream analyses, including antigenicity assessment and molecular docking studies. This careful filtering step is crucial in identifying precise vaccine candidates.

### 3.4 Transmembrane Helix Filtering

As part of the vaccine candidate refinement process, transmembrane helices were identified and excluded to ensure that only accessible extracellular proteins were retained. Transmembrane proteins often have regions embedded in the lipid bilayer or extending into the intracellular environment, making them less suitable for vaccine development due to limited immune accessibility [37]. Using the TMHMM v2.0 server, a total of 26 ORFs encoding extracellular proteins were identified after removing sequences containing transmembrane helices. Additionally, 36 ORFs corresponding to outer membrane proteins were retained for further analysis. This filtering ensured that the selected proteins were optimally positioned for immune recognition and interaction, fulfilling a critical criterion for subunit vaccine design [38].

### 3.5 ORF Similarity Search With Antigens Using BLAST

To identify potential antigenic ORFs with similarity to known *H. pylori* antigens, a BLAST search was performed using the NCBI BLAST tool (https://blast.ncbi.nlm.nih.gov/Blast.cgi). The refined ORFs from the previous filtering steps (26 extracellular and 36 outer membrane proteins) were compared against the non‐redundant protein database. The results showed significant homology with flagellin B [ *H. pylori* ] (GenBank: GAA9909008.1, Accession: GAA9909008), a 514‐amino‐acid protein, exhibiting 99.81% similarity, an E‐value of 0.0, and 100% query coverage. Based on the updated epitope analysis, 2 LBL, 6 HTL, and 6 CTL epitopes were selected from flagellin B. The entire CTB protein was incorporated as an adjuvant, and four additional CTL epitopes from urease B (UreB) were included to enhance cross‐presentation. By focusing on extracellular and outer membrane proteins, we narrowed the candidate pool to proteins with the highest potential for immune activation, while acknowledging that these predictions remain computational and require experimental validation to confirm their biological relevance.

### 3.6 ORFs Assessments and Antigen Selection

To identify the most promising antigens for vaccine development, we conducted a comprehensive assessment of the selected ORFs using four key bioinformatics tools. These evaluations ensured the exclusion of unsuitable ORFs and the prioritization of those with the highest potential for immunogenicity and safety. The antigenicity of each ORF was predicted using the VaxiJen v3.0 server (https://www.ddg‐pharmfac.net/vaxijen/VaxiJen/VaxiJen.html). Only ORFs with a VaxiJen score above the threshold value of 0.4 were considered antigenic and retained for further analysis. This step ensured the identification of ORFs capable of eliciting a strong immune response. Using the BLAST tool (https://blast.ncbi.nlm.nih.gov/Blast.cgi), ORFs were analyzed for homology with human and animal proteins. ORFs showing significant similarity to host proteins were excluded to minimize the risk of cross‐reactivity and autoimmunity, ensuring the specificity of the vaccine candidates. The AllerTOP v2.1 server (https://www.ddg‐pharmfac.net/AllerTOP/) was employed to evaluate the allergenicity of each ORF. Proteins predicted to be allergenic were excluded from the candidate pool, reducing the likelihood of adverse allergic reactions in future applications. The toxicity of the ORFs was predicted using ToxinPred (http://crdd.osdd.net/raghava/toxinpred/). This analysis ensured the exclusion of ORFs with toxic properties, further refining the candidate pool to include only safe proteins. Based on the combined results of these analyses, the top ORF identified as the most suitable candidate for vaccine development is presented in Table S1. This ORF demonstrated high antigenicity, no significant homology with host proteins, and was predicted to be non‐allergenic and non‐toxic.

### 3.7 CTL Epitopes Prediction and Assessment

After rigorous analysis using criteria such as antigenicity, immunogenicity, non‐allergenicity, and non‐toxicity, 10 CTL epitopes were retained. Among these, 6 epitopes were identified from flagellin B, and 4 were derived from urease B (UreB), which have been suggested to potentially contribute to cross‐presentation and CD8^+^ T‐cell responses. This selection process highlights their possible relevance in subunit vaccine design (Table 1), while emphasizing that these findings are computational predictions that require experimental confirmation.

**TABLE 1:** CTL epitopes identified from flagellin B and UreB.

Antigen	CTL epitopes	Immunogenicity	Antigenicity	Allergenicity	Toxicity
flagellin B	EASLDIQGR	0.904178	1.7235	Probable non‐allergen	Non‐toxic
	YTVNLRAVR	0.872263	1.6788	Probable non‐allergen	Non‐toxic
	HAVGVQNNR	0.969152	1.3683	Probable non‐allergen	Non‐toxic
	GVAEYTVNL	0.886788	1.0657	Probable non‐allergen	Non‐toxic
	ETNSQGIGA	0.472645	2.1122	Probable non‐allergen	Non‐toxic
	NSQGIGAGV	0.564916	1.8733	Probable non‐allergen	Non‐toxic
UreB	EVTSKPATK	0.937699	1.3578	Probable non‐allergen	Non‐toxic
	IGFKIHEDW	0.841469	1.326	Probable non‐allergen	Non‐toxic
	DSRIRPQTI	0.79924	1.4265	Probable non‐allergen	Non‐toxic
	IPTPQPVYY	0.995265	0.7004	Probable non‐allergen	Non‐toxic

### 3.8 HTL Epitopes Prediction and Assessment

Helper T lymphocyte (HTL) epitopes were predicted during the initial analysis and evaluated based on stringent criteria, including antigenicity, immunogenicity, non‐allergenicity, and non‐toxicity, to ensure the selection of the most promising candidates for vaccine development. After this rigorous assessment, 6 HTL epitopes were retained as optimal targets (Table S2).

### 3.9 LBL Epitopes Prediction and Assessment

Linear B lymphocyte (LBL) epitopes were initially identified through computational analysis and underwent a detailed evaluation based on key parameters such as antigenicity, non‐allergenicity, and non‐toxicity to ensure the selection of the most suitable candidates for vaccine development. Following this comprehensive screening process, 2 B‐cell epitopes were selected as the most promising candidates (Table 2).

**TABLE 2:** 2 B‐cell epitopes were selected as the most promising candidates.

LBL epitopes	Antigenicity	Allergenicity	Toxicity
NNISVTQVNVKAAESQIRDVDF	1.393	Probable non‐allergen	Non‐toxic
KAMDEQIK	1.2589	Probable non‐allergen	Non‐toxic

### 3.10 Vaccine Conformation

The vaccine was assembled, incorporating a total of 18 unique epitopes, comprising 10 cytotoxic T lymphocyte (CTL) epitopes (6 from flagellin B and 4 from urease B for cross‐presentation), 6 helper T lymphocyte (HTL) epitopes, and 2 linear B lymphocyte (LBL) epitopes. The CTLs, HTLs, and LBLs were connected using specific linkers: AAY for CTLs, GPGPG for HTLs, and KK for LBLs, as illustrated in Figure S2. Additionally, the entire cholera toxin B subunit (CTB) protein was incorporated as an adjuvant, attached to the N‐terminal of the vaccine construct via an EAAAK linker. The CTB adjuvant was selected for its role in enhancing immune responses, particularly by stimulating strong mucosal immunity, which is critical for gastrointestinal vaccines against *H. pylori* . CTB's ability to bind GM1 ganglioside receptors promotes robust antibody production and T‐cell activation, improving vaccine efficacy [39]. Thus, incorporating CTB as an adjuvant enhances immunogenicity, immune memory, and potential protection against *H. pylori* ‐induced gastric diseases [40]. The complete vaccine consisted of 406 amino acids.

### 3.11 Immunological Evaluation, Homology, and Physiochemical Properties of the Construct Vaccine

The constructed vaccine demonstrated an antigenic value of 1.0084 in VaxiJen, confirming its antigenic potential. It was also identified as non‐allergenic and non‐toxic, as indicated in Table 3. The BLAST search results revealed no significant homology between the final vaccine sequence and any human or animal proteins, including those found in species commonly used in preclinical studies, such as mice. This indicates that the vaccine protein is highly specific and minimizes the risk of autoimmunity or cross‐reactivity in the host. Various physiochemical traits of the vaccine were analyzed, revealing a number of amino acids of 406, a molecular weight of 43424.77 Da, a theoretical isoelectric point (pI) of 9.07, and a chemical formula of C1916H3012N554O587S7, with a total of 6076 atoms. The extinction coefficient at 280 nm in H2O was 39 435, with estimated half‐lives of 3.5 h in mammalian reticulocytes (in vitro), 10 min in yeast cells (in vivo), and over 10 h in *E. coli* (in vivo). The vaccine exhibited an instability index (II) of 23.51, indicating stability, an aliphatic index of 76.77, suggesting thermostability, and a grand average of hydropathicity (GRAVY) of −0.425, indicating hydrophilicity (Table S3). Regarding the secondary structure, the MLRC server identified 114 α‐helix residues (28.08%), 107 β‐strand residues (26.35%), and 185 random coil residues (45.57%) (Table S4).

**TABLE 3:** Immunological evaluated characteristics of the construct vaccine.

Immunological evaluated characteristic	Finding	Predicted result
Antigenicity	1.0084	Antigenic
Allergenicity	Non‐allergen	Non‐allergen
Toxicity	Non‐toxic	Non‐toxic

### 3.12 Tertiary Structure

**FIGURE 1:**
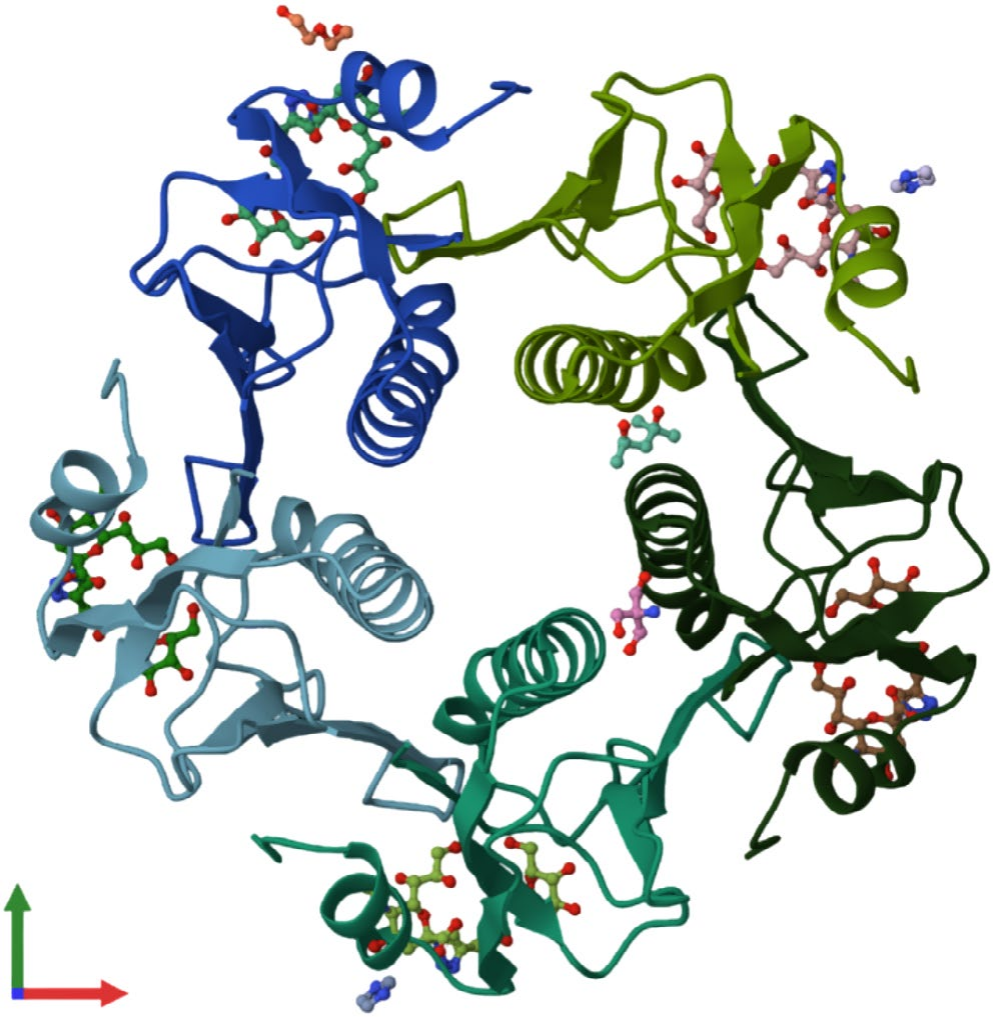
Tertiary structure of the designed vaccine.

**FIGURE 2:**
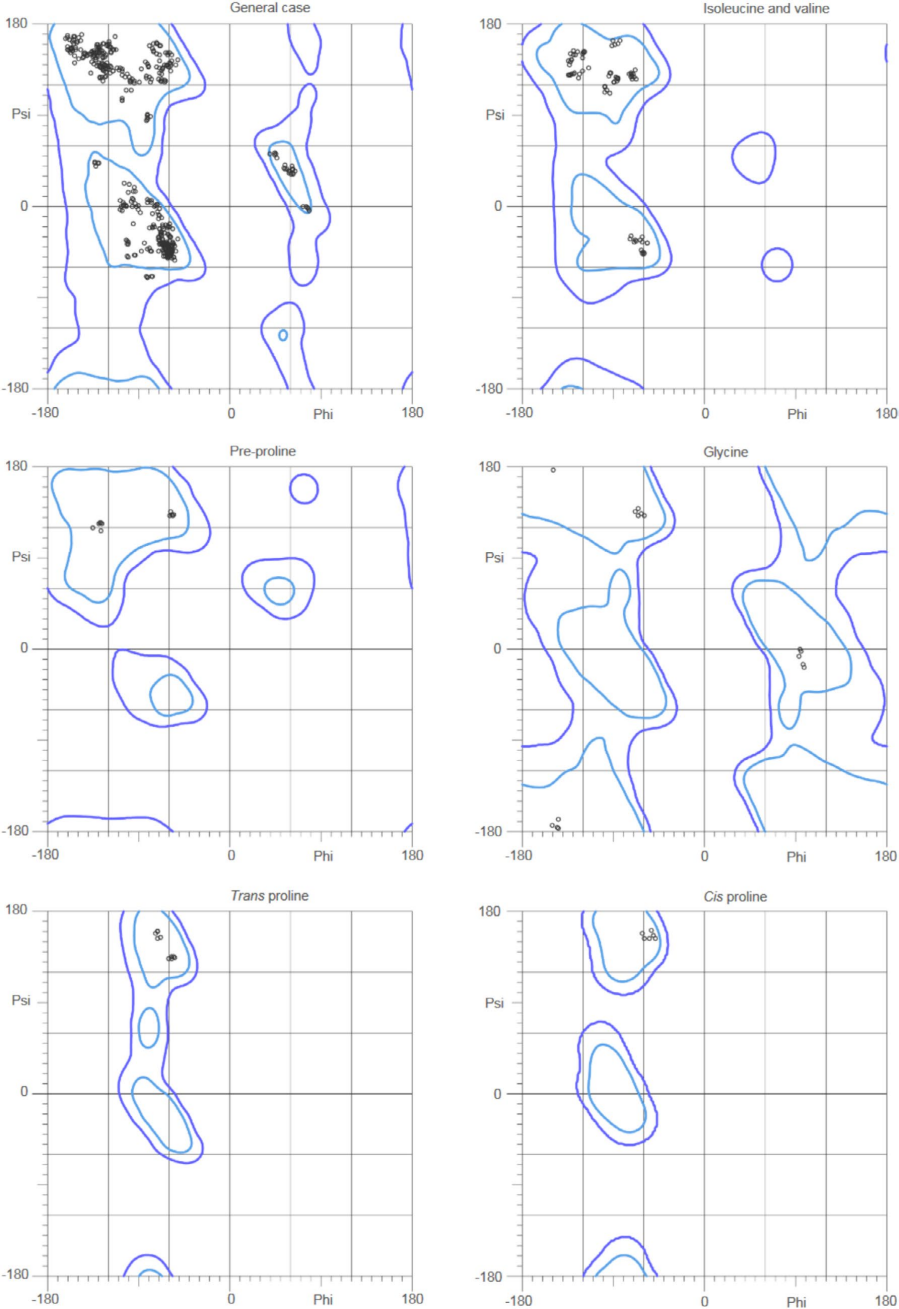
Ramachandran analysis. 99.1% (525/530) of all residues were in favored (98%) regions.

In this study, homology modeling was performed using the Phyre2 server to generate the three‐dimensional (3D) structure of the protein. The top model was based on the template 5lzgD_ from the Protein Data Bank (Figure 1), corresponding to the cholera enterotoxin subunit B from *Vibrio cholerae O1 biovar El Tor str. N16961*. The model achieved 100.0% confidence with 25% coverage, where 103 residues (25% of the total sequence) were modeled based on the single highest‐scoring template. Further refinement using MolProbity revealed favorable geometrical properties based on the Ramachandran analysis of 5lzg (PDB), model 1: 99.1% (525/530) of all residues were in favored regions (goal > 98%), 100.0% (530/530) of all residues were in allowed regions (goal > 99.8%), and there were no outliers (Figure 2). These validations confirmed the model's quality, supporting its use for subsequent analyses and potential applications. The details are summarized in Table S5.

### 3.13 Molecular Docking Exploration

Molecular docking analysis was conducted using HDOCK [33], which integrates a hybrid docking algorithm for protein–protein and protein‐ligand interactions, targeting Toll‐like receptor 2 (TLR2) and Toll‐like receptor 5 (TLR5). The docking simulation generated 100 potential docked positions for each receptor, with results ranked by their respective docking scores, confidence scores, and ligand root‐mean‐square deviations (RMSD) in angstroms (Å). For TLR2, Table 4 summarizes the top 10 docking poses, highlighting their docking and confidence scores alongside the ligand RMSD values, with Model 1 exhibiting a docking score of −250.74, a confidence score of 0.8823, and a ligand RMSD of 159.64 Å. For TLR5, Table 4 presents the top 10 docking poses, with Model 1 displaying a docking score of −309.05, a confidence score of 0.9601, and a ligand RMSD of 122.03 Å. The TLR5 Model 1 was selected as the optimal docking model (Figure S3) due to its lower (more negative) docking score and higher confidence score, indicating a stronger binding affinity and greater reliability of the predicted pose.

**TABLE 4:** Summary of the top 10 models in HDOCK server targeting TLR2 and TLR5.

Receptor	Model	Rank	Docking score	Confidence score	Ligand rmsd (Å)
TRL2	Model 1	1	−250.74	0.8823	159.64
	Model 2	2	−247.93	0.8764	158.33
	Model 3	3	−247.14	0.8747	162.89
	Model 4	4	−243.41	0.8662	79.22
	Model 5	5	−239.23	0.8563	78.38
	Model 6	6	−236.74	0.8500	160.91
	Model 7	7	−234.76	0.8449	67.74
	Model 8	8	−233.08	0.8405	88.95
	Model 9	9	−232.96	0.8401	89.03
	Model 10	10	−232.48	0.8388	96.42
TRL5	Model 1	1	−309.05	0.9601	122.03
	Model 2	2	−297.46	0.9502	154.83
	Model 3	3	−295.91	0.9487	229.35
	Model 4	4	−294.26	0.9471	165.17
	Model 5	5	−293.34	0.9462	163.14
	Model 6	6	−285.73	0.9379	229.37
	Model 7	7	−284.19	0.9361	151.69
	Model 8	8	−283.13	0.9348	163.65
	Model 9	9	−280.43	0.9314	100.09
	Model 10	10	−277.54	0.9276	155.15

The docking score reflects the energy of interaction, with more negative values suggesting enhanced binding affinity, a critical factor for the designed vaccine's interaction with TLR5, a preferred receptor known for recognizing flagellin [29]. The confidence score further supports the robustness of the TLR5 Model 1 prediction. However, the elevated RMSD values (159.64 Å for TLR2 and 122.03 Å for TLR5) warrant discussion. These higher RMSDs are attributable to the inherent structural flexibility of large protein–protein complexes, a phenomenon noted in protein–protein docking studies where conformational changes during interaction are common [41]. Pierce et al. also indicate that RMSD values exceeding 20 Å and even up to 100 Å or more can occur due to the dynamic nature of protein structures, particularly in rigid‐body docking approaches like HDOCK, suggesting that such deviations do not necessarily invalidate the model if supported by favorable docking and confidence scores [42]. Thus, the selected TLR5 Model 1 remains a viable candidate, with its high docking score (−309.05) and confidence (0.9601) outweighing the RMSD concern, though experimental validation (e.g., in vitro binding assays) is recommended to confirm the predicted interaction. This model underscores the potential effectiveness of the designed ligand in targeting TLR5, aligning with its biological role in immune activation.

## 4 Discussion

*H. pylori* infection is a major public health challenge with no approved vaccine or effective treatment currently established [43]. Many patients do not know that they have acute or chronic gastritis and precancerous lesions and will not receive therapy until the symptoms become exacerbated [44].

Triple and quadruple bismuth‐containing therapies are effective standard treatments but have limitations. These challenges include patient adherence, increased emergence of antibiotic resistance, and the potential of reinfection. The applications of current therapeutic protocols are substantially limited by the rapid breakdown antibiotics experience under acidic conditions, their limited ability to cross the gastric mucus layer and biofilm, and their inadequate absorption by gastric epithelial cells required to eliminate intracellular *H. pylori* [45]. Therefore, the advancement of earlier and better screening techniques is needed to allow for prevention and eradication of *H. pylori* across populations and to achieve concomitant treatment options for current infections in patients [46]. So, there is significant importance for the development of an effective vaccine against *H. pylori* . Despite research spanning 30 years, there are no licensed prophylactic vaccines against *H. pylori* , and many clinical studies published have only completed phase I [14].

In recent years, scientists have considered the possibility of creating multi‐epitope vaccines by focusing on various bacterial antigens to enhance immune response. Multi‐epitope vaccines are characterized by quicker development timelines, reduced costs, and enhanced safety profiles, making them very promising [47]. Many multi‐epitope‐based vaccines have been developed in the literature targeting individual antigens, which may be more subject to reduced efficacy due to antigenic variation and mutations [48]. In contrast, our research employs a more robust approach by combining epitopes from multiple proteins, including Urease B and Flagellin B. This method reduces the possibility that changes to one protein will give the bacterium the ability to escape immune recognition.

Extensive evidence suggests that carefully selected linear B cell epitopes, especially those that are accessible on the surface and of functional importance, can elicit targeted and neutralizing antibody responses to bacterial pathogens. Research conducted on bacterial toxins, such as *Bacillus anthracis* protective antigen, has identified linear neutralizing B‐cell epitopes and led to the generation of antibodies that are capable of preventing infection [49]. In addition, vaccination with linear B cell epitopes derived from *H. pylori* urease resulted in significantly increased levels of neutralizing antibodies able to inhibit urease activity and significantly reduce bacterial colonization in a mouse model [50].

*H. pylori* infections have multiple virulence factors that contribute to their pathogenicity, including the cytotoxicity‐associated gene (CagA), the vacuolating cytotoxin A (VacA), urease, flagella and associated proteins, the hopQ adhesin gene, the sialic acid‐binding adhesin (SabA), the outer inflammatory protein (OipA), and the blood group antigen‐binding adhesin (BabA) [45].

Urease contributes to the colonization of the gastric mucosa and is also a major virulence factor by damaging host tissue through ammonia production and perturbing host immune responses [51, 52]. In *H. pylori* , the UreB gene is responsible for the production of urease B, a component of urease, which directly interacts with TLR2 on immune cells. This engagement represents the initial step in the activation of the NLRP3 inflammasome pathway, leading to the expression of the NLRP3 inflammasome. The interaction is crucial for triggering the inflammasome and subsequently facilitating the maturation of interleukin‐1β (IL‐1β), thus playing a significant role in regulating innate immune responses [53, 54]. In addition, UreB contains epitopes recognized by cytotoxic T lymphocytes (CTLs), which are critical for adaptive immunity, and TLR2 signaling initiated by UreB drives tolerogenic dendritic cell programming resulting in limited Th1 responses and increased regulatory T cells that support ongoing infection with *H. pylori* [53, 55].

Flagellins of *H. pylori* , in particular Flagellin B, are crucial for motility, which is essential for the ability of *H. pylori* to colonize gastric mucosal surfaces and significantly together increase pathogen virulence [10]. Flagellated bacteria cause the host to recognize the flagellin with TLR5 as a pathogen‐associated molecular pattern which activates the innate immune response [36]. Recruitment of TLR5‐dependent immune cells via the CCL2/CCR2 pathway is critical to the development of functional CD4+ T‐cell‐mediated immunity, which helps control bacterial colonization and decreases the degree of infection [56].

Although the computational approaches used in this work provide a more efficient and less costly way to produce a vaccine, it is important to understand these limitations. The predictions and designs require thorough experimental testing. Investigations should confirm the immunogenicity of the chosen epitopes in vitro, and subsequent in vivo experiments are necessary to assess the vaccine's effectiveness and safety in various population groups [57]. The findings from this research highlight the potential advantages of using immunoinformatics for theoretical vaccine design. However, several challenges remain and must be carefully addressed before any transition from computational predictions to experimental and practical applications (e.g., stability, effectiveness, and duration) [28, 57].

## 5 Conclusion

This study illustrates the application of an immunoinformatics‐driven approach in theoretically designing a multi‐epitope subunit vaccine candidate targeting *H. pylori* . The vaccine construct integrates 10 cytotoxic T lymphocyte (CTL) epitopes (6 from flagellin B and 4 from urease B), 6 helper T lymphocyte (HTL) epitopes, and 2 linear B lymphocyte (LBL) epitopes, linked with specific linkers (AAY for CTLs, GPGPG for HTLs, and KK for LBLs), and incorporates the cholera toxin B subunit (CTB) as a mucosal adjuvant via an EAAAK linker at the N‐terminal. This configuration, comprising 406 amino acids with a molecular weight of 43424.77 Da, demonstrated a predicted antigenic profile (VaxiJen score of 1.0084), non‐allergenic and non‐toxic properties, and favorable physiochemical characteristics, including stability (instability index 23.51) and hydrophilicity (GRAVY −0.425). The tertiary structure, modeled with 100.0% confidence using the Phyre2 server and validated by MolProbity (99.1% favored and 100.0% allowed Ramachandran regions), supports the theoretical structural integrity of the construct. Molecular docking analysis with TLR5, yielding a docking score of −309.05 and a confidence score of 0.9601, suggested stronger binding affinity compared to TLR2, highlighting TLR5 as a plausible target for immune activation.

These findings provide a preliminary computational basis for a vaccine candidate with the potential to elicit immune responses against *H. pylori* ‐associated diseases, including gastric cancer. However, the study remains entirely in silico, and rigorous experimental validation through in vitro and in vivo studies is essential to assess efficacy, safety, and immunogenicity. Additionally, future investigations will be required to optimize dosing regimens, explore administration routes (particularly mucosal delivery), and evaluate long‐term immune memory. Only if validated through experimental research could such a vaccine contribute to reducing the global burden of *H. pylori* ‐related pathologies. This work therefore represents a computational starting point rather than a translational endpoint, aligning with peer review expectations for rigorous in silico design followed by experimental follow‐up.

## Author Contributions

**Aytak Vahdat Khajeh Pasha:** writing – original draft, visualization, investigation, formal analysis, data curation, software, project coordination. **Mohammad Esfandiyari:** writing – review and editing, validation, formal analysis, investigation, software. **Alireza Parnian:** writing – original draft, supervision, writing – review and editing, validation, software. **Mohammad Mahboubi‐Rabbani**, **Maryam Bayanati:** writing – original draft, validation, funding acquisition, supervision, project administration, conceptualization, writing – review and editing.

## Funding

The authors have nothing to report.

## Ethics Statement

The authors have nothing to report.

## Conflicts of Interest

The authors declare no conflicts of interest.

## Supporting information


**Figure S1:** Diagram of total procedure of this research,
**Figure S2:** Graphical map of the designed multi‐epitope vaccine construct.
**Figure S3:** Molecular docking between the vaccine (ligand) and the TLR5 (receptor).

**Table S1:** Selected antigen properties
**Table S2:** 6 HTL epitopes were retained as optimal targets.
**Table S3:** Physiochemical properties of the construct vaccine.
**Table S4:** Secondary structure of the construct vaccine.
**Table S5:** Summary of protein geometry.

## Data Availability

The data that support the findings of this study are available on request from the corresponding author. The data are not publicly available due to privacy or ethical restrictions.
