# Supplementary material for: Immunoinformatics‐Based Multi‐Epitope Vaccine Targeting Helicobacter Pylori
Source: Cancer Rep (Hoboken). 2025 Dec 28;9(1):e70441. doi: 10.1002/cnr2.70441 (PMC12745834; doi:10.1002/cnr2.70441)
Supplement: Supplementary file 1 — Figure S1: Diagram of total procedure of this research, Figure S2: Graphical map of the designed multi‐epitope vaccine construct. Figure S3: Molecular docking between the vaccine (ligand) and the TLR5 (receptor). [file CNR2-9-e70441-s002.docx]

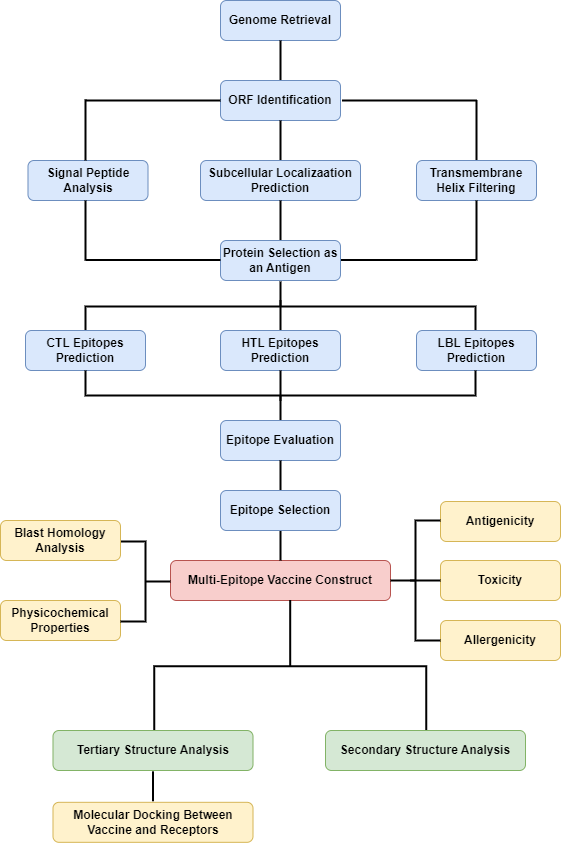


Figure 1S .Diagram of total procedure of this research


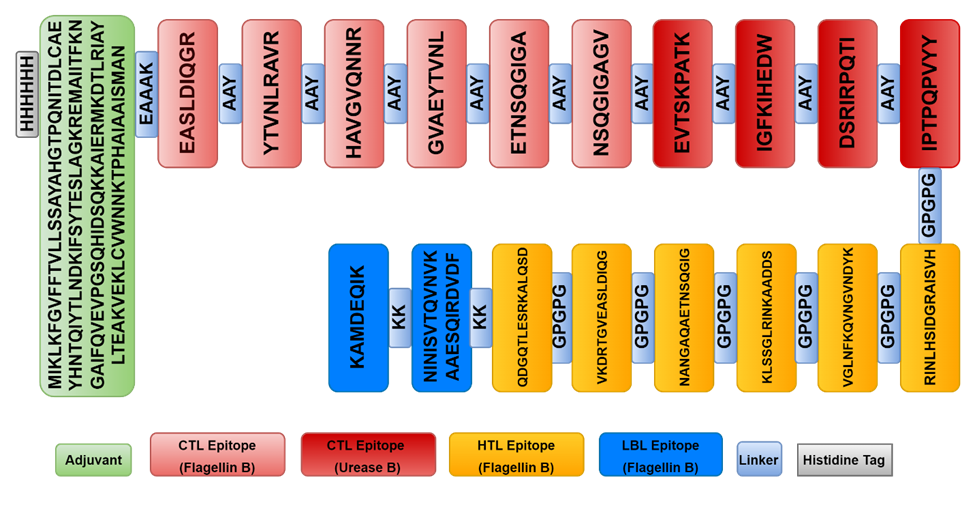


Figure 2S. Graphical map of the designed multi-epitope vaccine construct


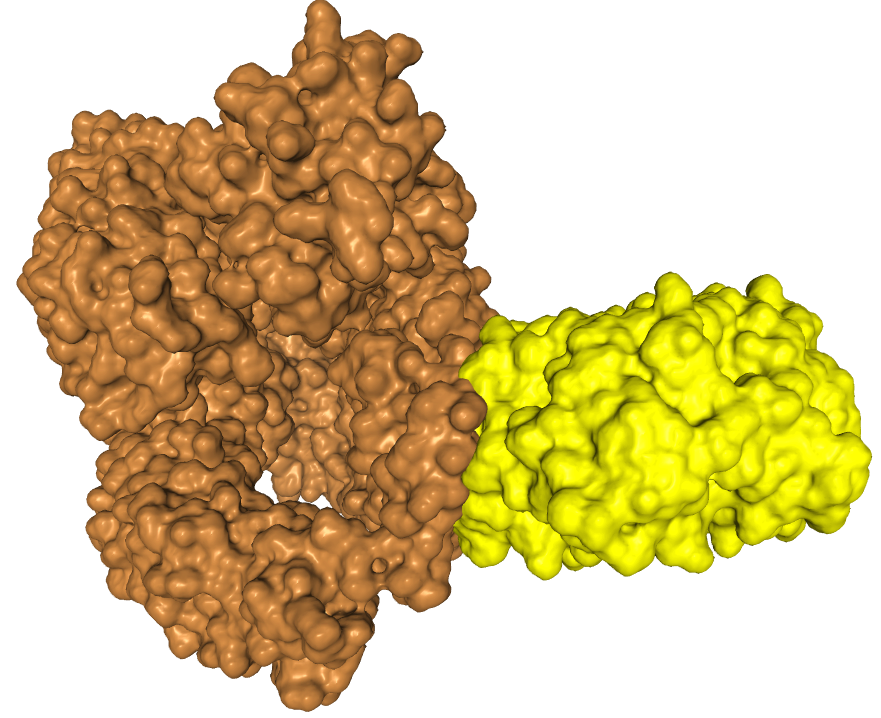

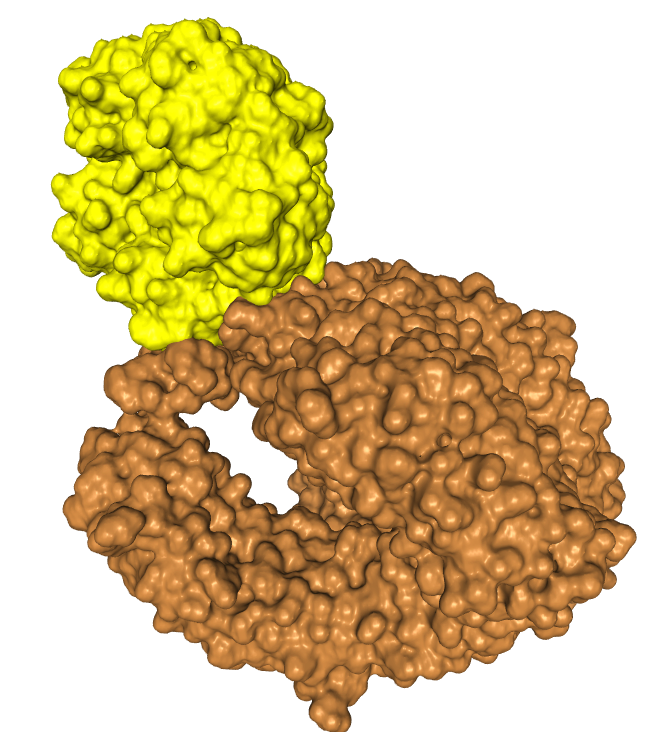


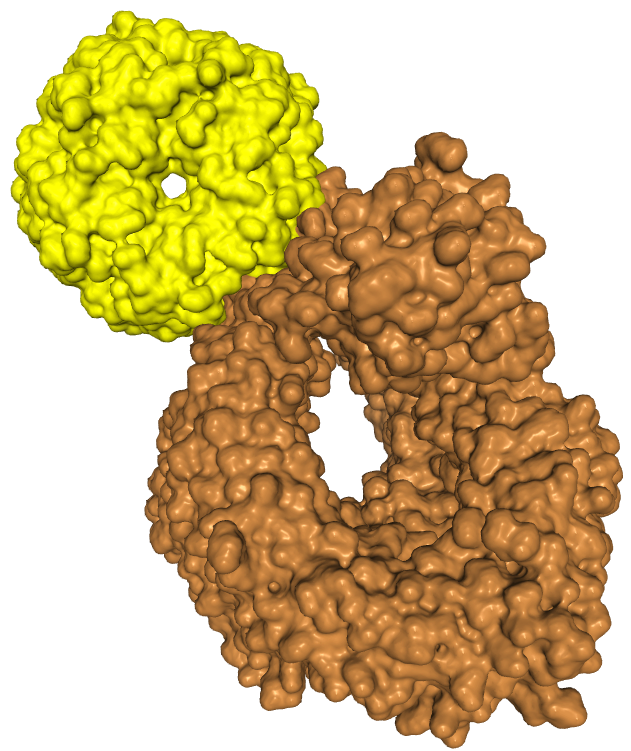

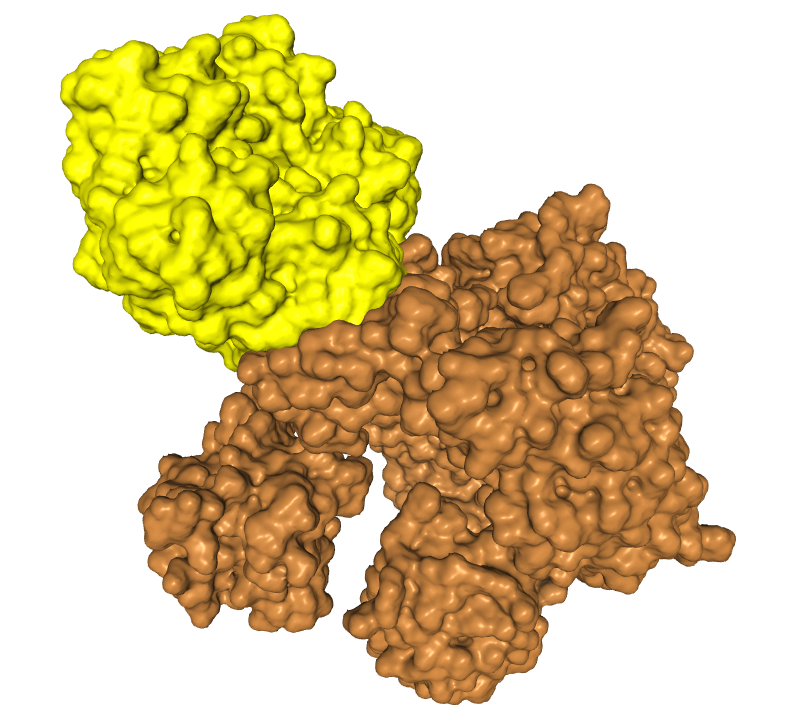


Figure 3S. Molecular docking between the vaccine (ligand) and the TLR5 (receptor).
