# Supplementary material for: Immunoinformatics‐Based Multi‐Epitope Vaccine Targeting Helicobacter Pylori
Source: Cancer Rep (Hoboken). 2025 Dec 28;9(1):e70441. doi: 10.1002/cnr2.70441 (PMC12745834; doi:10.1002/cnr2.70441)
Supplement: Supplementary file 2 — Table S1: Selected antigen properties Table S2: 6 HTL epitopes were retained as optimal targets. Table S3: Physiochemical properties of the construct vaccine. Table S4: Secondary structure of the construct vaccine. Table S5: Summary of protein geometry. [file CNR2-9-e70441-s001.docx]

Table 1S. Selected antigen properties

| Antigenicity Score | Blast | Allergenicity | Toxicity |
| --- | --- | --- | --- |
| 0.7844 | OK | Probable non-allergen | Non-toxic |

Table 2S. 6 HTL epitopes were retained as optimal targets

| HTL Epitopes | Immunogenicity | Antigenicity | Allergenicity | Toxicity |
| --- | --- | --- | --- | --- |
| RINLHSIDGRAISVH | 0.652362 | 2.0647 | Probable non-allergen | Non-toxic |
| VGLNFKQVNGVNDYK | 0.794597 | 1.3256 | Probable non-allergen | Non-toxic |
| KLSSGLRINKAADDS | 0.757101 | 1.0773 | Probable non-allergen | Non-toxic |
| NANGAQAETNSQGIG | 0.131142 | 2.1339 | Probable non-allergen | Non-toxic |
| VKDRTGVEASLDIQG | 0.344043 | 1.6648 | Probable non-allergen | Non-toxic |
| QDGQTLESRKALQSD | 0.537753 | 1.2498 | Probable non-allergen | Non-toxic |

Table 3S. Physiochemical properties of the construct vaccine.

| Characteristics | Finding | Remark |
| --- | --- | --- |
| Number of amino acids | 406 | Suitable |
| Molecular weight | 43424.77 Da | Average |
| Theoretical pI | 9.07 | Base |
| Chemical formula | C_1916_H_3012_N_554_O_587_S_7_ |  |
| Total number of atoms | 6076 |  |
| Extinction coefficient (at 280 nm H2O) | 39435 |  |
| Estimated half-life (mammalian reticulocytes, in vitro) | 3.5 h |  |
| Estimated half-life (yeast-cells, in vivo) | 10 min |  |
| Estimated half-life (E. coli, in vivo) | >10 h |  |
| Instability index (II) of vaccine | 23.51 | Stable |
| Aliphatic index of vaccine | 76.77 | Thermostable |
| Grand average of hydropathicity (GRAVY) | -0.425 | Hydrophilic |

Table 4S. Secondary structure of the construct vaccine

| Features | Amino Acids | Percentage |
| --- | --- | --- |
| α-helix | 114 | 28.08% |
| β-strand | 107 | 26.35% |
| Random coils | 185 | 45.57% |

Table 5S. Summary of protein geometry.

| Geometrical Property | Value (raw count) | Value (percentage) | Goal | Assessment |
| --- | --- | --- | --- | --- |
| Poor rotamers | 2 | 0.43% | <0.3% | Caution |
| Favored rotamers | 449 | 96.77% | >98% | Caution |
| Ramachandran outliers | 0 | 0.00% | <0.05% | Good |
| Ramachandran favored | 500 | 99.01% | >98% | Good |
| Rama distribution Z-score | 0.13 ± 0.34 | | Z score < 2 | Good |
| Cβ deviations >0.25Å | 1 | 0.19% | 0 | Caution |
| Bad bonds | 1/4339 | 0.02% | 0% | Caution |
| Bad angles | 7/5887 | 0.12% | <0.1% | Caution |
